# Digital biomarkers and sex impacts in Alzheimer’s disease management — potential utility for innovative 3P medicine approach

**DOI:** 10.1007/s13167-022-00284-3

**Published:** 2022-06-06

**Authors:** Robbert L. Harms, Alberto Ferrari, Irene B. Meier, Julie Martinkova, Enrico Santus, Nicola Marino, Davide Cirillo, Simona Mellino, Silvina Catuara Solarz, Ioannis Tarnanas, Cassandra Szoeke, Jakub Hort, Alfonso Valencia, Maria Teresa Ferretti, Azizi Seixas, Antonella Santuccione Chadha

**Affiliations:** 1 Altoida Inc., Houston, TX USA; 2 Women’s Brain Project, Guntershausen, Switzerland; 3 Chione GmbH, 8122 Binz, Switzerland; 4 Memory Clinic, Department of Neurology, Second Faculty of Medicine, Charles University and Motol University Hospital, Prague, Czech Republic; 5 Bayer, NJ USA; 6 Dipartimento Di Scienze Mediche E Chirurgiche, Università Degli Studi Di Foggia, Foggia, Italy; 7 Barcelona Supercomputing Center, Plaça Eusebi Güell, 1-3, 08034 Barcelona, Spain; 8 Global Brain Health Institute, Dublin, Ireland; 9 Centre for Medical Research, Faculty of Medicine, Dentistry and Health Science, University of Melbourne, Melbourne, Australia; 10 International Clinical Research Center, St Anne’s University Hospital Brno, Brno, Czech Republic; 11 ICREA - Institució Catalana de Recerca I Estudis Avançats, Pg. Lluís Companys 23, 08010 Barcelona, Spain; 12 Department of Psychiatry and Behavioral Sciences, University of Miami Miller School of Medicine, Miami, FL 33136 USA

**Keywords:** Alzheimer’s disease, Digital biomarkers, Sex, Classifier, Predictive preventive personalized medicine (PPPM)

## Abstract

Digital biomarkers are defined as objective, quantifiable physiological and behavioral data that are collected and measured by means of digital devices. Their use has revolutionized clinical research by enabling high-frequency, longitudinal, and sensitive measurements. In the field of neurodegenerative diseases, an example of a digital biomarker-based technology is instrumental activities of daily living (iADL) digital medical application, a predictive biomarker of conversion from mild cognitive impairment (MCI) due to Alzheimer’s disease (AD) to dementia due to AD in individuals aged 55 + . Digital biomarkers show promise to transform clinical practice. Nevertheless, their use may be affected by variables such as demographics, genetics, and phenotype. Among these factors, sex is particularly important in Alzheimer’s, where men and women present with different symptoms and progression patterns that impact diagnosis. In this study, we explore sex differences in Altoida’s digital medical application in a sample of 568 subjects consisting of a clinical dataset (MCI and dementia due to AD) and a healthy population. We found that a biological sex-classifier, built on digital biomarker features captured using Altoida’s application, achieved a 75% ROC-AUC (receiver operating characteristic — area under curve) performance in predicting biological sex in healthy individuals, indicating significant differences in neurocognitive performance signatures between males and females. The performance dropped when we applied this classifier to more advanced stages on the AD continuum, including MCI and dementia, suggesting that sex differences might be disease-stage dependent. Our results indicate that neurocognitive performance signatures built on data from digital biomarker features are different between men and women. These results stress the need to integrate traditional approaches to dementia research with digital biomarker technologies and personalized medicine perspectives to achieve more precise predictive diagnostics, targeted prevention, and customized treatment of cognitive decline.

**Supplementary Information:**

The online version contains supplementary material available at 10.1007/s13167-022-00284-3.

## Introduction

### Digital biomarkers in personalized medicine

In the last two decades, biomarkers have been increasingly utilized as measurable indicators of a subject’s state in clinical research and clinical practice [1, 2]. Advances in sensor technology and increasing omniscience of research-grade devices have paved the way for digital biomarker-based technologies to rise in prominence. The difference between traditional and digital biomarkers is that the latter are collected via digital devices and can be collected outside of traditional clinical settings. The digital devices collecting these biomarkers can include wearables, implantables, ingestible devices, and smartphones and tablets [3, 4]. Examples of digital biomarkers are objective consumer-grade data such as voice [5], temperature [6], activity [7], gait [8], blood oxygen [9], heart rate [10], touch [11], and augmented reality [12, 13], all collected via mobile and wearable technologies. As opposed to standard clinical measures, digital biomarkers enable high-frequency, longitudinal, and objective measurements, largely independent of the clinical rater. Digital biomarkers can continuously monitor patients to assess therapy response and disease progression without the need for clinical assessment [4, 14]. Moreover, they often exhibit higher sensitivity than traditional clinically used methods, enabling early predictive diagnostics by identifying patients at risk of overt clinical disease [15].

### Applications in Alzheimer’s disease

Digital biomarkers can make significant contributions to the assessment and diagnosis of neurodegenerative diseases, specifically Alzheimer’s disease [16]. Alzheimer’s disease (AD) is a lethal neurodegenerative disease, causing a progressive loss of neurocognitive functions, eventually leading to dementia and death. The underlying pathological changes start to occur in the brain as early as midlife with a long “silent” preclinical phase [17]. The accumulation of a toxic aggregated peptide, amyloid-beta (Aβ), in the brain is one of the hallmark biological indicators of ongoing AD pathology [18]. The combination of MCI with Aβ is typically considered an early indicator of Alzheimer’s disease, i.e., prodromal AD [19]. Predictive diagnostics using AD biomarkers in the presymptomatic or oligosymptomatic (MCI) stage, followed by targeted preventions and treatment personalized to those individuals considered high risk, are increasingly considered to represent the best chance at successful AD management [20]. The importance of early diagnosis is recently being emphasized with the introduction of treatments targeted against AD pathophysiological hallmarks, such as aducanumab. Compared to traditional methods of detecting AD (e.g., neuropsychological testing, genetic testing, imaging [21]), digital biomarkers are particularly well suited for testing early stages of AD because they can detect subtle behavioral, cognitive, motor, and sensory changes in the early stages of AD.

Although digital biomarkers show great promise, it is essential to consider that multiple factors can affect biomarker state and their predictive value, including demographic, genetic, and phenotypic aspects. Among these factors, sex has emerged as a crucial factor in several disorders [16, 22] and notably in AD [23]. Considering such differences, it is vital to increase the predictive value of any set of biomarkers. For instance, stratification of data by sex has been shown to increase predictivity of a polygenic hazard score for AD [24]. Similarly, in the ABIDE (Alzheimer’s biomarkers in daily practice) study, which developed risk models for AD based on CSF biomarkers, models that stratified the markers by sex were more predictive than those that did not [25].

### Interactions between sex and digital biomarkers

Sex is progressively recognized as a crucial source of AD heterogeneity and a promising target for personalized care in AD. Considering sex in predictive diagnostics as well as targeted prevention and sex-specific considerations in clinical trials [26] enables better accuracy in diagnostic and prognostic stratification [24, 25, 27] and may precipitate more successful targeted AD drug development. A number of digital instruments that have proven able to help with diagnosis of AD or MCI are now available, a few examples being MemTrax [28], CNS Vital Signs [29], CANTAB-PAL [30], and CAMCOG-CAT [31] (see [32] for a review). A recent promising example of a digital biomarker-based technology for early diagnosis of AD is Altoida Inc.’s digital medical application [33]. The company Altoida Inc. has developed an application, in research known as the Neuro-Motor Index (NMI), which leverages a smartphone- or tablet-based activity battery using augmented reality (AR) and finger motor tasks to simulate activities of instrumental daily living (iADLs). From the digital biomarker data collected during these activities, the device uses artificial intelligence (AI) to help predict an individual’s conversion from MCI to dementia, including dementia due to AD. In Buegler 2020 [33], Altoida demonstrated that the device is capable of detecting conversion to AD with a 94% ROC-AUC, which is comparable to or moderately better than traditional biomarker-based approaches [25]. Additionally, the tablet-based activity allows home use and eliminates invasive procedures such as lumbar puncture, which could enable application of predictive diagnostics to larger groups of patients. In turn, it could allow for more wide-spread specific prevention in at-risk groups, ease of use in potential future clinical trials, and personalized application of future disease-modifying treatments. Digital approaches for prevention might be envisioned as predictive algorithms that consider a number of variables, risk factors, and protective factors, on top of objective measures of brain function, to estimate risk and potential progression. It has been estimated that about 40% of dementias are caused by modifiable risk factors [34], emphasizing the importance of early prediction. If risk is identified, digital tools like iADL might be put in place to monitor functions and the effect of preventative actions. In this context, as different risk profiles as well as progression of cognitive decline exist for men and women, considering sex differences might help us making such predictive algorithms more precise.

### Working hypothesis in the framework of predictive, preventive, and personalized medicine (3PM/PPPM)

To date, it is unknown whether sex differences exist in digital biomarkers for AD. If there are indeed sex differences in digital biomarkers, their consideration is informative in interpreting the biomarker’s diagnostic and prognostic value, as well as for potential biomarker use in clinical trials. In this work, we explore the ability to use digital biomarker data, captured by Altoida’s application, to identify sex-based neurocognitive performance. To this end, we trained a classifier using all digital features captured by Altoida’s application to distinguish between male and female subjects. We show here for the first time that this sex classifier could correctly predict sex in 75% of the cases, indicating that the digital biomarkers captured by Altoida’s application are able to capture significant differences in the neurocognitive performance signature of biological males and biological females. We next investigated the effects of age and different stages of AD on the success of the sex classifier. Finally, we investigated the relative importance of each digital biomarker feature and describe here the most discriminative features. Our work informs future sex-based personalization of digital biomarker algorithms, with implications for AD risk stratification, early diagnostics, and prevention.

## Methods

### Data collection

We used a combination of clinical and population data, collected and provided by Altoida Inc. The clinical data (*n* = 438 data points) consists of controlled tests of elderly (50 +) subjects with known biological and cognitive biomarkers (e.g., MCI, amyloid-beta (Ab) + , Ab-, AD). We used the dataset described as “New validation study” (ClinicalTrials.gov Identifier: NCT02843529) for this work, and the original purpose of which was to evaluate the performance of Altoida’s application as an adjunctive tool for diagnosing AD. The subjects were classified into the clinical groups of healthy (normal cognition), MCI, or AD dementia according to the internationally recognized National Institute on Aging- Alzheimer’s Association (NIA-AA) criteria [35]. The inclusion/exclusion criteria as well as further details on classification have previously been described in detail in publication [33] as well as in the details of the clinical trial (ClinicalTrials.gov Identifier: NCT02843529). The data was collected in Roma, Brescia, and Naples in Italy, with *n* = 60, males = 14, females = 46 data points, in Corfu, Thessaloniki in Greece with *n* = 166, males = 48, females = 118 data points, and in Spain, Barcelona in Spain with *n* = 212, males = 98, females = 114 data points). The inclusion and exclusion criteria were identical for each country.

The population sample consists of a group of middle-aged cognitively healthy Japanese subjects (*n* = 130). The inclusion criteria for participation were age 20–50 and self-assessed cognitively healthy (i.e., no known cognitive disorders). The subjects received no stipend for participation and permission for scientific studies was provided by accepting the terms and conditions of Altoida Inc. All subject information was anonymized and de-identified. Beyond the digital biomarkers collected by the Altoida application, no further biomarkers were recorded for this population sample. For both datasets, the subject’s sex was self-reported. All subjects (of both groups) performed multiple test sessions using Altoida’s application. See Table 1 for a distribution of the subjects by sex and AD progression.

**Table 1:** Distribution of data points over sex and AD progression. *MCI* mild cognitive impairment, *AD* Alzheimer’s disease, *Ab* amyloid-beta

	Healthy male	Healthy female	MCI Ab − male	MCI Ab − female	MCI Ab + male	MCI Ab + female	AD male	AD female	Total male	Total female
Clinical data	104	201	16	26	35	43	5	8	160	278
Population data	94	36	0	0	0	0	0	0	94	36

### Definition of sex and gender

The terms sex and gender are not synonymous. Whereas biological sex is driven by the expression of sexual chromosomes and sexual hormones, gender is related to the socio-cultural construct of being a man or a woman in a given society [36]. Here, we use the term “sex” to refer to the individuals in the study identifying their sex as “man” or “woman” at enrollment.

### Digital biomarkers

**Fig. 1:**
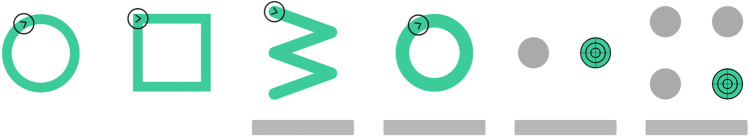
The motoric functioning tasks in the Altoida test. These are executed one after another. Using their index finger of their dominant hand, from left to right, the task is to (1) draw a circle, (2) draw a square, (3) draw a rotated W shape within 7 s, (4) draw as many circles as possible within 7 s, (5) tap the highlighted buttons (left, right, left, right, etc.), and (6) tap the highlighted button as fast as possible, the buttons highlight at random

**Fig. 2:**
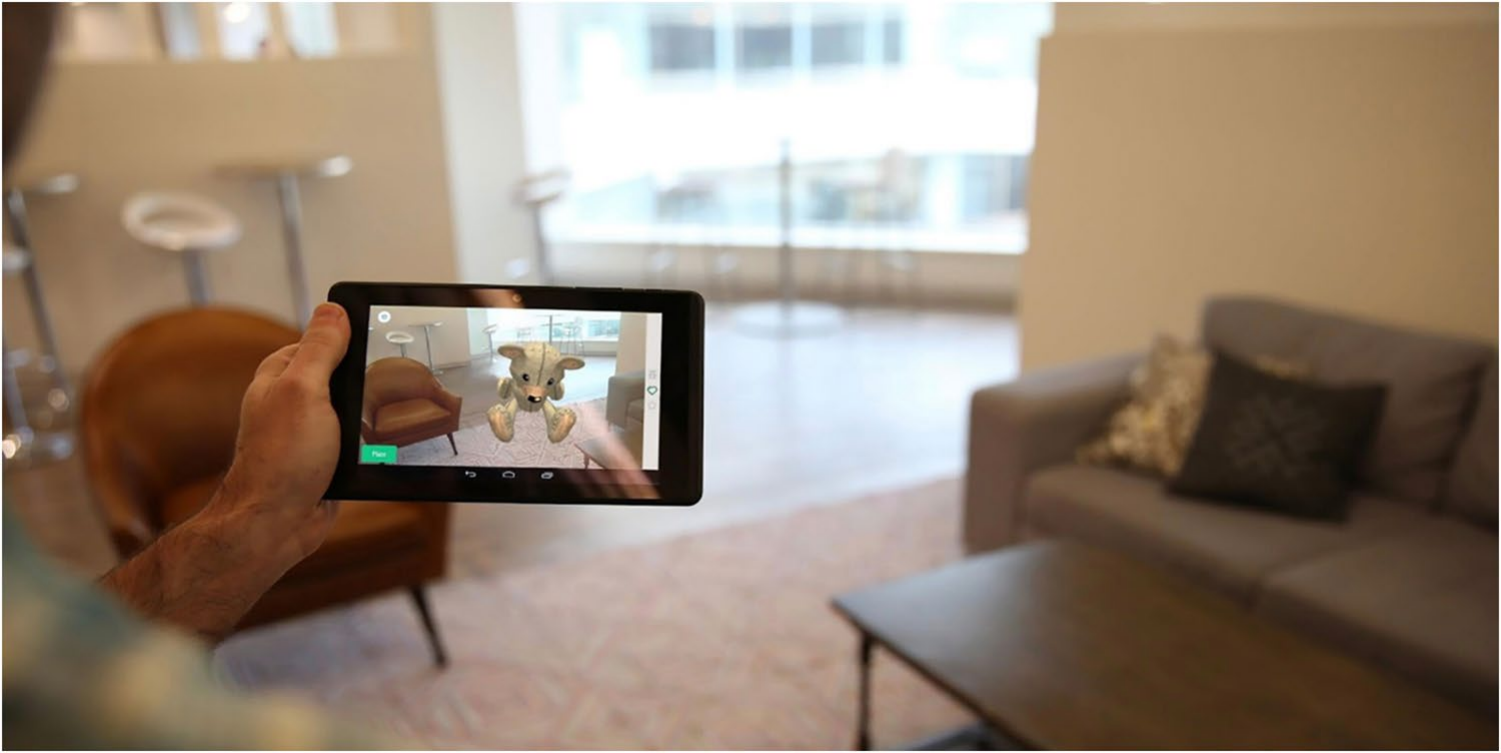
Illustration of the augmented reality (AR) task in the Altoida test. During the AR test, the subject is asked to place and find three virtual objects in the room. To do so, the subject is required to walk around the room holding a tablet or smartphone device in front of him/her. While doing so, the camera of the device records the environment and displays it back to the user on the screen, augmented with virtual objects (in this illustration, a teddy bear). The user needs to place the objects on flat surfaces and later recall their position by walking back to that location

For this work, we repurposed data from Altoida’s application which collects digital biomarkers for neurocognitive function measurement and predictive diagnosis of AD [33]. Altoida’s application collects digital biomarker data for detecting early onset AD. While holding a tablet or smartphone device, the subject is asked to perform a series of motor functioning tasks and two augmented reality (AR) tasks. In the motor functioning tasks, the subject is required to draw shapes and tap on the (touch)screen using the finger of their dominant hand (see Fig. 1 for an illustration of all the motor functioning tasks). In one of the AR tasks, the subject is asked to place three virtual objects in a small space (approximately 3 m × 3 m or 2 m × 4 m) and afterward find them again. The AR task is performed by navigating around the space with the tablet or smartphone in both hands (see Fig. 2). During these tasks, the handheld device collects telemetry and touch data from the built-in sensors, enabling profiling of hand micro-movements, screen touch pressures, walking speed, navigation trajectory, cognitive processing speed, and additional proprietary inputs.

A single test session using Altoida’s application consists of two batches of motor tasks and two AR tasks. After a subject completes all tasks, the recorded digital biomarker data from the onboard electronics sensors is bundled and securely and anonymously uploaded to a server for further processing. In previous work, machine learning was either used to classify subjects as healthy or at risk of AD (Buegler et al. 2020). In this work, we examined digital biomarker data from test sessions using Altoida’s application to see if they demonstrated sex differences, expressed by the capacity of results to inform a sex classifier. Provided the data of multiple subjects, machine learning can be used to detect patterns.

### Machine learning

From the onboard electronics sensors, we extracted 793 digital biomarker features describing various cognitive, functional, and physiological characteristics of each subject. These features include response times, eye-hand coordination precision, fluctuations in the telemetry (accelerometer and gyroscope) data, Fourier analysis of the telemetry data, step detection, and additional proprietary data. Based on the digital biomarker feature data from a selection of healthy subjects, we trained a sex classifier to distinguish males from females. For the classification, we used the XGBoost algorithm [37] with sex as the target variable.

### Performance evaluation

We applied stratified fivefold grouped cross-validation to estimate the generalization performance of the sex classifier. We grouped data points by subject to ensure that multiple data points of a single subject were all in the same fold (either training or testing), preventing learning bias. For our sex classifier, we report ROC-AUC (receiver operating characteristic area under curve), accuracy, and precision averaged over the 5 cross-validation testing folds. To assess the performance of the sex classifier on different age groups and different stages of AD, we trained 9 additional classifiers (10 in total) each using different random subsets of the data. We report the ROC-AUC averages, with SD, over the 10 different classifiers as the main performance criteria.

### Model explainability

We used the Shapley Additive exPlanations (SHAP) [38] method to better understand the predictions made by the sex classifier. The SHAP method allocates to each feature of a classifier a game-theoretical value representing the contribution of that feature towards the classification targets. The sign of the SHAP values indicates the direction of the contribution and the magnitude of the SHAP value indicates the importance. For our classifier, negative SHAP values contribute to classifying as female, positive numbers towards male. SHAP values have an additive property meaning they can be summed together to provide the feature contribution of a group of features [39].

## Results

### Sample characteristics and data collection

Table 2 describes our data characteristics for the entire sample and stratified by sex, with univariate comparisons. Our data consists of 568 subjects combined from two datasets, a clinical dataset [33] and a healthy population dataset (see Methods “Data collection”). The clinical dataset consists primarily of female subjects (*n* = 314, 55%) with mean age of 67.6 years (see supplementary Table S1), and the healthy population dataset consists primarily of male subjects with mean age of 35 years (see supplementary Table S1). Subjects were distributed over several stages of the AD clinical continuum, namely healthy (77%), MCI (amyloid-beta negative) Ab- (7%), MCI Ab + (14%), and dementia due to AD (2%) as reported by clinical assessment. All subjects with MCI or AD come from the clinical dataset. We obtained one or more trials of digital biomarker data collection using Altoida’s application, providing us with a median distribution of 2 data points per subject. To counter the imbalance from multiple data points per subject and combining two demographically different datasets, we stratified all analysis by dataset, sex, and number of data points. This ensures that we have exactly the same number of data points from each sex and from each study (clinical and population).

**Table 2:** Data characteristics. *P*-value is calculated using two-sided *t*-test for age, chi square for status, and the Mann–Whitney rank test for the number of data points per subject

			**Men**	**Women**	**Total**	***p*-value**
Population	Clinical data	*N* (%)	160 (63%)	278 (89%)	438 (77%)	< 0.001
	Population data	*N* (%)	94 (37%)	36 (11%)	130 (23%)	
Age		Mean (*SD*)	56.9 (17.4)	62.7 (12.8)	60.1 (15.3)	< 0.001
Status	Healthy	*N* (%)	198 (78%)	237 (75%)	435 (77%)	0.786
	MCI ab −	*N* (%)	16 (6%)	26 (8%)	42 (7%)	
	MCI ab +	*N* (%)	35 (14%)	43 (14%)	78 (14%)	
	AD	*N* (%)	5 (2%)	8 (3%)	13 (2%)	
Number of NMI trials (data points)		*N* (%)	948 (52%)	859 (48%)	1807	-
Number of NMI trials (data points) per subject		Median (*IQR*)	2 (4)	2 (5)	2 (3)	< 0.001

### Training and testing of a sex classifier

**Fig. 3:**
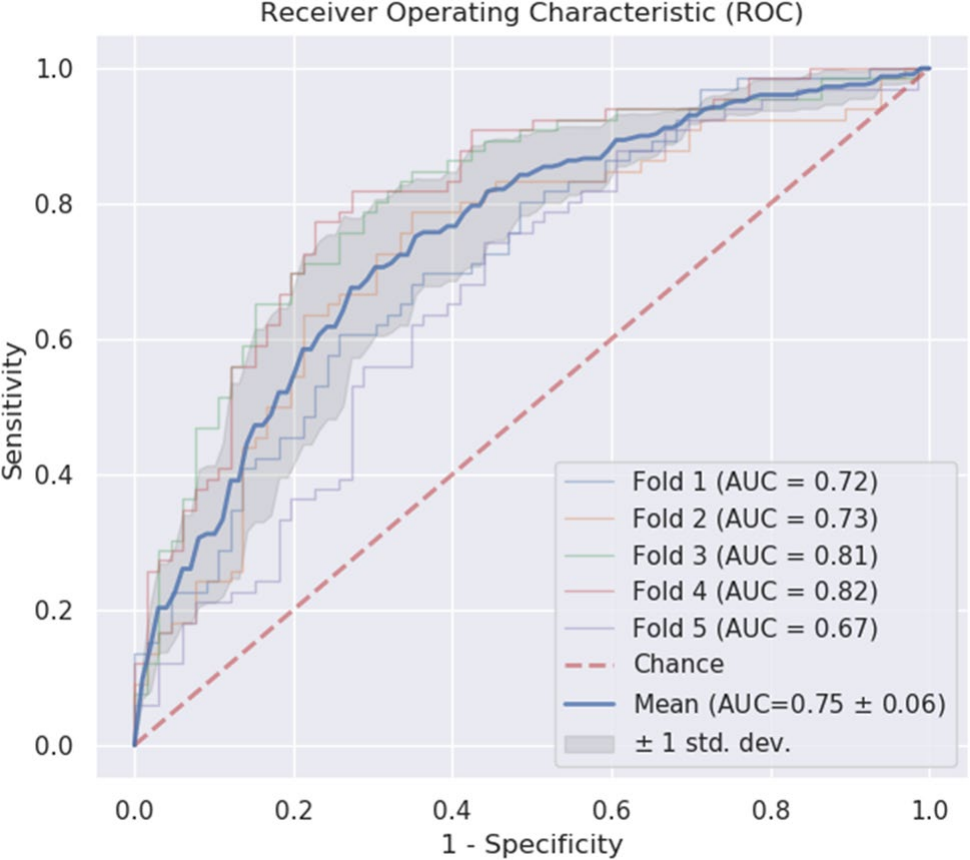
Receiver operating characteristic (ROC) curve with five-fold cross-validation results for the digital biomarker sex classifier

We trained an XGBoost classifier [37] with sex as the target variable. Although there is an imbalance in the subject’s demographics, our intention was to build a single sex classifier and base our further findings on this classifier. Separate classification statistics for the Japanese population data and cohort subjects can be found in the supplementary material Sup. Figures 1 and 2. For our single classifier, to handle the imbalances in the subjects’ demographics, we selected training data from a combination of four groups: clinical trial healthy males, clinical trial healthy females, population data healthy males, and population data healthy females. In addition, due to imbalance in the number of data points per subject, we decided to take 165 data points at random from the pool of data points of each of the four previously mentioned groups to prevent over-representation of data of one of the groups. In total, we selected four times 165 (660 in total) data points from our database as training data for a single sex classifier. In essence, this is a random sub-sample of our entire dataset, stratified by sex and study (clinical and population). Our classifier was then trained on a set of 660 data points (of 348 cognitively healthy subjects), which we used in both training and testing. Each datapoint corresponds to a set of 793 digital biomarker features collected using Altoida’s application. The classifier’s generalizing performance was assessed by fivefold cross-validation (Fig. 3) giving an AUC of 0.75 (*STD* ± 0.06), which is considered “fair” [40]. The classifier exhibits a similar performance in accuracy (0.71 ± 0.05) and precision (0.71 ± 0.06).

### Performance of sex classifier on different disease sub-groups

To assess the performance of the sex classifier at different stages of the AD clinical continuum, we applied the previously trained sex classifier to MCI-amnestic subjects without amyloid-beta in their cerebrospinal fluid (MCI/Aβ-; 42 subjects), MCI-amnestic subjects with Aβ + (MCI/Aβ + ; 78 subjects), and subjects diagnosed with AD dementia (13 subjects). To make the results more robust, we trained 9 additional sex classifiers using 9 different randomly sampled (sampled with replacement) subsets of our data. That is, we used the same stratified approach as discussed earlier and again sampled four times 165 data points to in total randomly select 660 data points. With such an approach, the number of subjects per classifier varies slightly, but the number of data points stays constant. Having trained the 9 additional sex classifiers on healthy data, we then applied all 10 to the sub-groups of MCI and AD subjects. In total, we therefore trained 10 sex classifiers on cognitively healthy subjects.

**Fig. 4:**
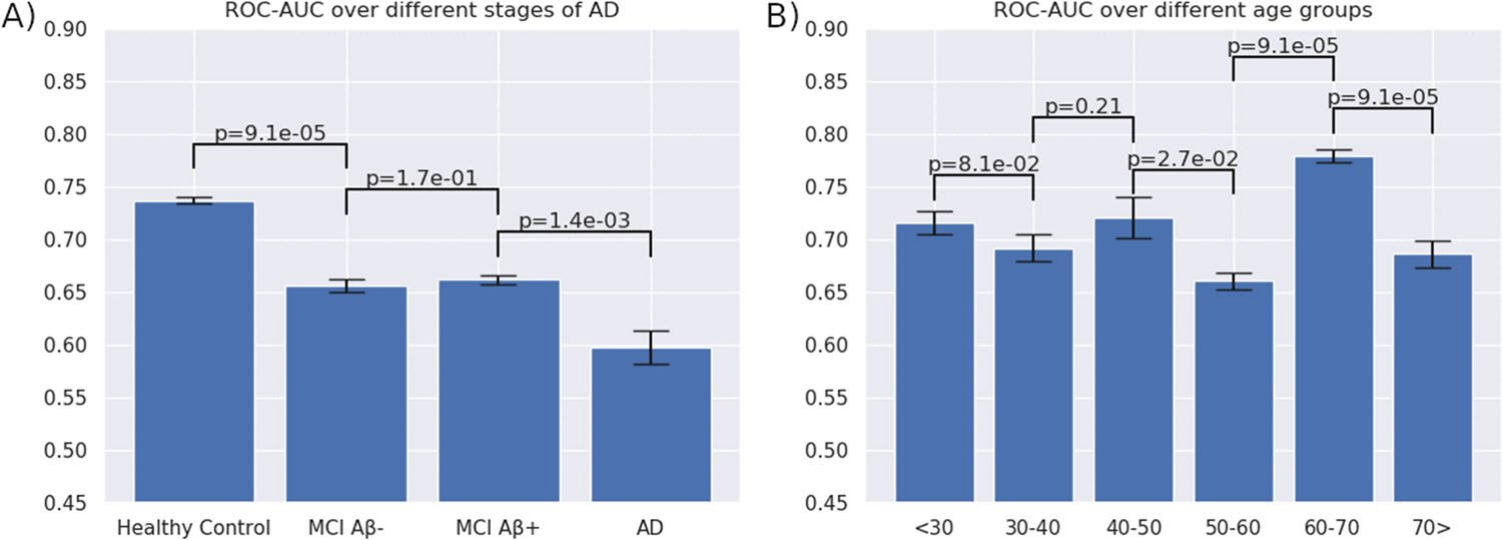
Receiver operating characteristics (ROC) area under curve (AUC) for different subgroups. **A** Comparison over different stages of Alzheimer’s progression. **B** Comparison over different age groups in the healthy population. Error bars show standard error of the mean (SEM). *P*-values are computed using the Mann Whitney *U* test

Figure 4A shows the average ROC-AUC performance of 10 sex classifiers for each of these sub-groups. Comparisons between subgroups are reported using a Mann Whitney *U* test [41]. The sex classifier performed best on healthy subjects with an average ROC-AUC score of 74%, slightly below the performance of the single classifier presented earlier. Performance difference between healthy and MCI-amnestic subjects is significant with (*P* < 0.001). Both MCI subgroups score at ROC-AUC 66%, with no significant difference between MCI Aβ- and MCI Aβ + (*P* = 0.17). Performance on the sex classifier in the AD subgroup was the lowest with an ROC-AUC of 60%, again scoring significantly lower than both MCI/Aβ- (*P* < 0.001) and MCI/Aβ + (*P* < 0.001).

### Performance of sex classifier on different age subgroups

The differences in sex classification between the various stages of AD might be due to an imbalance in age distribution across the subgroups. In particular, since women were overall older in our datasets, we also studied the effect of age on the sex classifier.

We used the cross-validation results of each of the ten sex classifiers to investigate the performance over the various age groups in the healthy population. We grouped all healthy subjects in age classes 10 years-wide and computed the sex classification performance in each of these subgroups. Figure 4B reports average sex classification performance per age group. ROC-AUC scores varied across age groups, with overall significant differences between the age groups. Most age groups lie close to the group average of 71% ROC-AUC, except the 60–70 year old group, which has a significantly higher value at 78% ROC-AUC. This is possibly related to our sample distribution which peaks at the age of 67–69 years for the clinical dataset (see additional material Table 1), with a small difference in distribution between males and females, as such, there is a slight imbalance in demographics within the 60–70 year group to which the ROC-AUC might be overly sensitive.

### Feature contribution in the sex classifier

We computed a SHAP value for each of the 793 digital biomarker features of our primary sex classifier to investigate which of the 793 features are the most relevant in the sex classifier. These values are calculated over the set of healthy subjects and illustrate the feature importance of the sex classifier as constructed using the same healthy subjects.

**Fig. 5:**
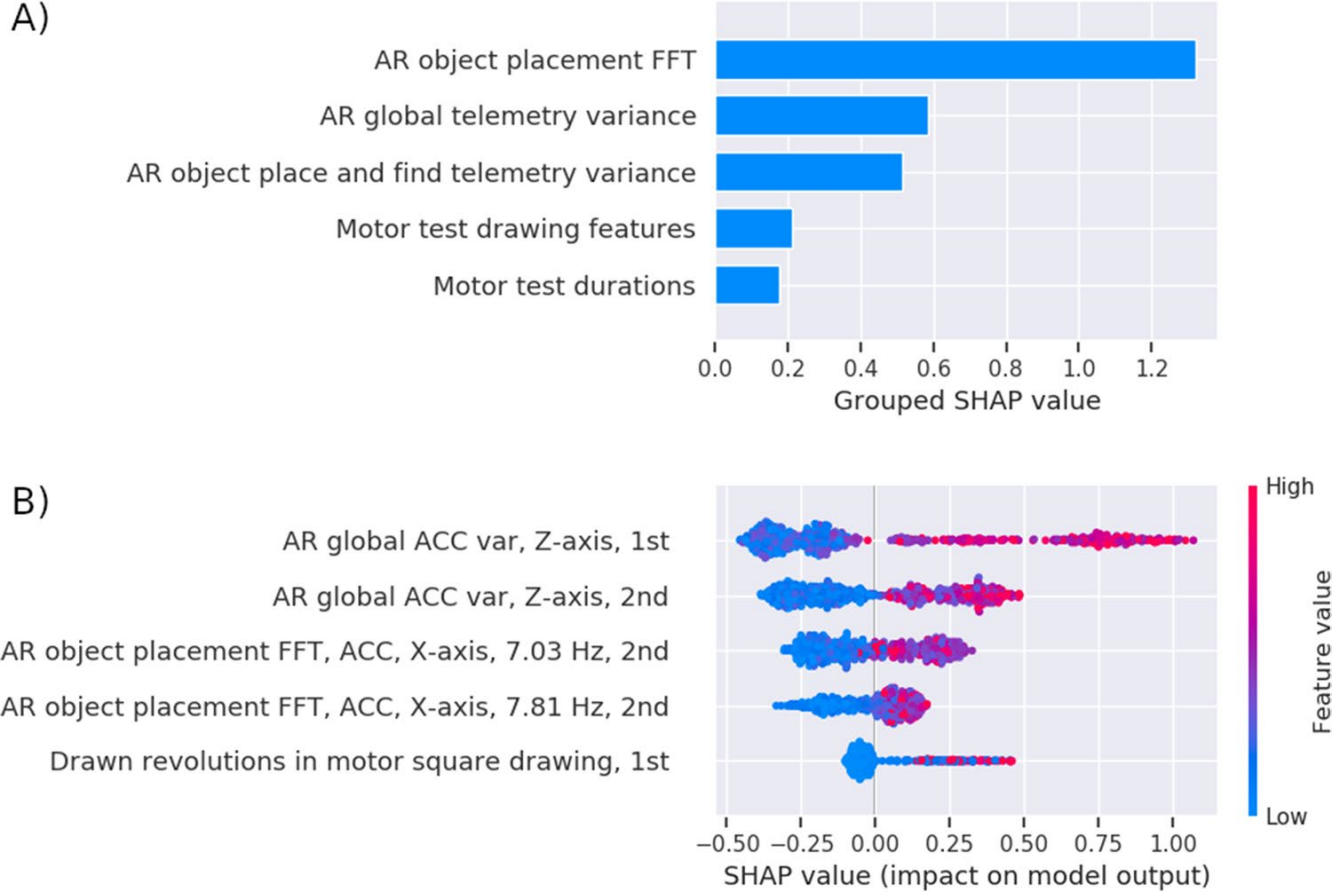
Feature importance of the sex classifier in healthy individuals. **A** The top five feature groups according to the SHAP method. Each bar represents the summed SHAP value of the features in that feature group. **B** A feature value SHAP distribution plot for the top five contributing features. Subject specific SHAP values were computed for each datapoint in the classifier training data. For each feature, we then plot for each datapoint a dot with the feature value of that datapoint, with the dot color coded by the relative feature value. The position of each dot on the SHAP value *x*-axis represents the magnitude and the direction of the contribution of that specific feature value of that specific datapoint towards classifying as female (− 1) or male (+ 1). Acronyms in the plots are augmented reality (AR), fast Fourier transform (FFT), SHapley Additive exPlanations (SHAP), accelerometer (ACC), variance (var), first part of a single test (1st), or second part of a single test (2nd)

Figure 5A shows a grouping of features that were ranked as having the highest overall contribution in the sex classifier. The primary contributing group of features is named the AR object placement fast Fourier transform (FFT). This group consists of a set of frequency magnitudes obtained by performing an FFT on the measured accelerometer and gyroscope signal over 1.28 s before placing a virtual object in the AR test. These features could therefore be interpreted as steady hand micro tremors. The second most important group of digital biomarker features is the AR global telemetry variance. The global telemetry variance is the variance in the accelerometer and gyroscope signal over the entire duration of the AR task. It could be interpreted as coarse scale hand motion variance. The third group, AR object place and find telemetry variance is similar to the previous group except that this takes the variance of the signal 1.28 s before placing and finding a virtual object. The fourth group “Motor test drawing features” considers the speed and accuracy of the subject while drawing various patterns with the index finger. The “Motor test duration” measures how long the user spent reading the instructions and performing the motor tests.

Figure 5B shows a SHAP distribution plot for the top five standalone highest contributing features. In this figure, we used SHAP to perform an ontology analysis of the features, as it allows to identify the individual contribution of each of the features. The top two contributing features are the AR global accelerometer variance in the *Z*-axis of both rounds of the AR task. These two features belong to the group of AR global telemetry variance of Fig. 5A. On a smartphone and tablet device, the *Z*-axis is the axis perpendicular to the screen with negative values pointing downwards to the floor. Since the device is handheld, this feature measures the accelerometer variance in up and down motion during the AR tasks. The third and fourth most important features are frequency magnitudes during object placement, belonging to the top group of Fig. 5A. The most important frequencies seem to be in the range of 7–8 Hz in the accelerometer’s *x*-axes, which is the axes to the left and right when holding a device. The fifth most important feature measures the number of revolutions the user drew in the motor square drawing test. It belongs to the group of motor test drawing features. The color coding shows that for each of the top five features, if the relative feature value is high (red), it indicates a contribution to classifying as male. If the value is low (blue), it indicates contribution to classifying as female.

### Feature differences in male, female, healthy, and MCI subjects

In the SHAP values in previous section, we observed that higher acceleration variances are more indicative of males and lower variances are more indicative of females (see Fig. 5B). Since these are the top performing features, in theory, this means that this relationship of high values for males, low for females, should be (at least somewhat) distorted in the group of MCI and AD subjects. In this section, we use histograms to gain a better understanding of why MCI and AD subjects score lower on our general sex classifier.

**Fig. 6:**
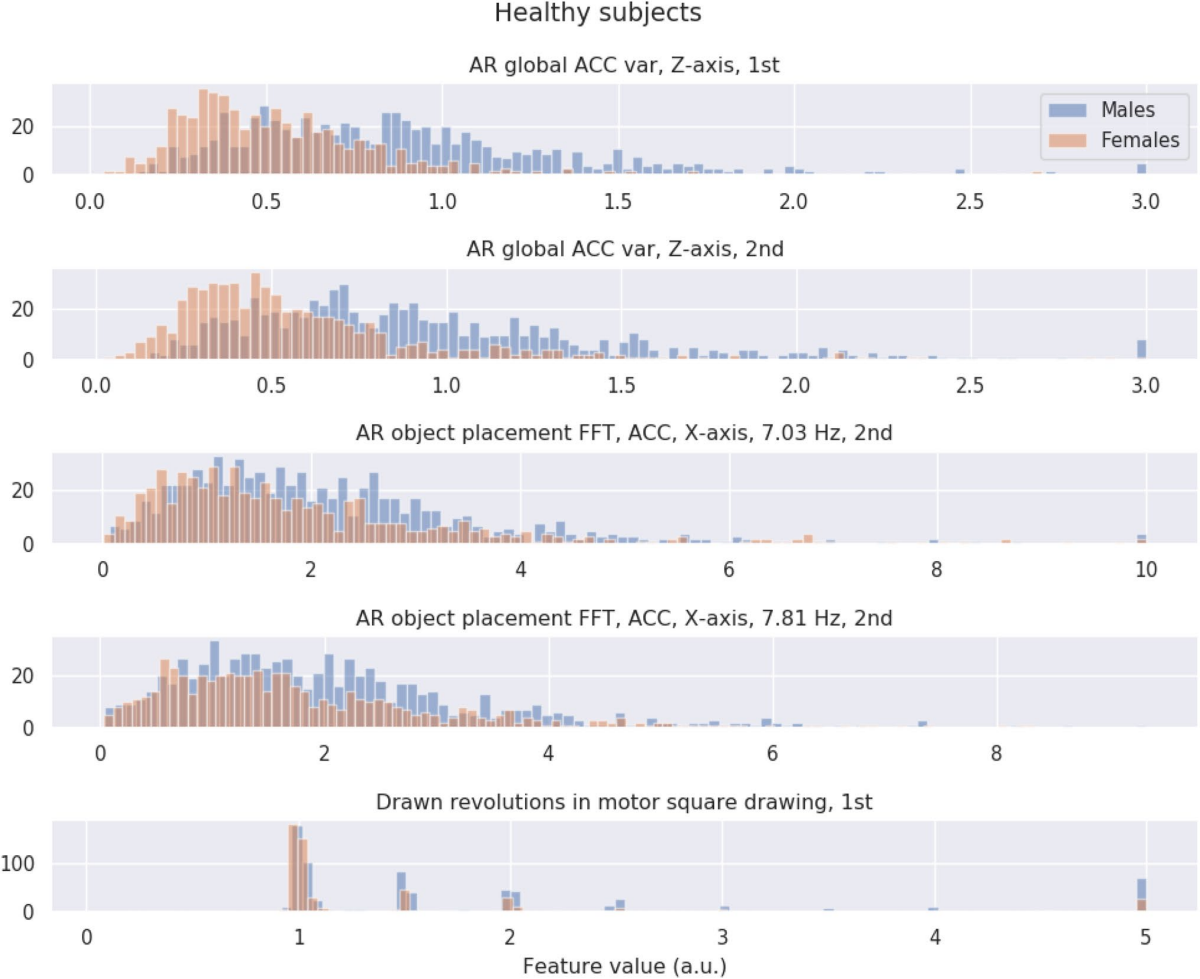
Comparative histograms of the top five contributing features (according to the SHAP results in Fig. 5) for the group of healthy subjects, with male data in blue and female data in red. Acronyms are augmented reality (AR), fast Fourier transform (FFT), accelerometer (ACC), variance (var), first part of a single test (1st), or second part of a single test (2nd)

**Fig. 7:**
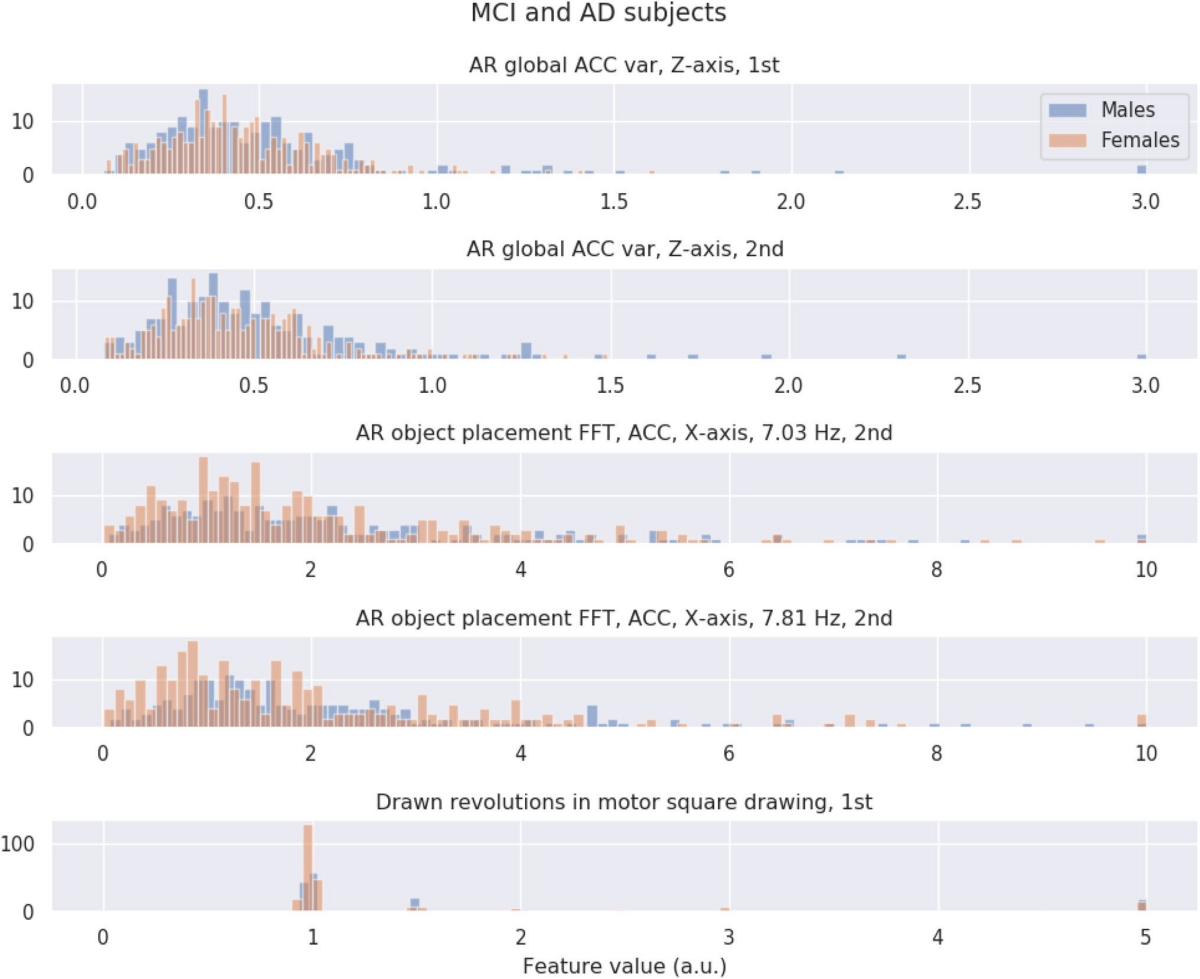
Comparative histograms of the top five contributing features (according to the SHAP results in Fig. 5) for the combined group of MCI and AD subjects, with male data in blue and female data in red. Acronyms are augmented reality (AR), fast Fourier transform (FFT), accelerometer (ACC), variance (var), first part of a single test (1st), or second part of a single test (2nd)

For both the group of healthy subjects and the group of MCI (including AD) subjects, we created a set of histograms showing the feature values of males and females for the top five performing features as indicated by SHAP. Figure 6 shows for the healthy subjects, for each of the top five features, a histogram of feature values for both the males (in blue) and the females (in red). We observe that the distribution of values is typically higher for the males and lower for the females, thereby confirming the SHAP predictions. Figure 7 shows the same set of histograms but then for the group of MCI and AD subjects. We observe that the distribution of feature values is highly similar between the males and the females with the distributions largely overlapping. Comparing Figs. 6 to 7, we further observe that the distribution for females remains largely unchanged, but that males in the MCI group now display lower feature values.

## Discussion

Neurodegenerative diseases and dementia are a field of study that shows high promise for application of precision and personalized medicine. Currently, clinical trials of drugs for treatment of AD have a very high degree of failure, suggesting that individual factors should be taken into account both in searching new treatments and in designing clinical trials [42]. Biological sex is one such factor; a recent meta-analysis showed that the proportion of women enrolled in clinical trials for AD is lower than in the general AD population, and that it correlates with severity of symptoms in the enrolled population [43]. An appraisal of the impact of sex on cognitive decline and treatment response in Alzheimer is pivotal to build personalized treatment tailored to the unmet clinical needs of the patients.

In this paper, we tested the hypothesis that sex differences exist in the digital biomarkers collected by Altoida’s application for detection of early AD. To this end, we developed a classifier which can predict the sex of the subject using digital biomarkers collected by Altoida’s application through a series of AR and motor activities. The sex classifier was built using 793 digital biomarker features and trained on a set of 348 healthy subjects with 660 data points. The classifier achieved a fair discriminatory ability in healthy subjects, with an ROC-AUC of 0.75. The high performance is noteworthy, as the digital biomarker features collected by Altoida’s application are devoid of demographic information. This result therefore shows that healthy males and females display detectable and unexpected neurocognitive performance signature differences when evaluated using Altoida’s application. In the field of predictive diagnostics, the result is striking as it suggests that overlooking the complex interaction between sex and predictive digital biomarkers can potentially impair our ability of early disease detection or of devising methods for targeted prevention of cognitive decline.

The discriminatory ability decreased when the classifier was externally validated on a set of patients in the AD continuum, from early stages (*MCI*, *AUC*: 0.66) to full-blown AD dementia (*AUC*: 0.60). Interestingly, the classifier had the same performance in MCI patients positive or negative for Aβ. Performance dropped significantly when validated with a small subset of patients with overt AD dementia. It is interesting that the drop in performance occurs gradually, with an initial decrease in MCI which progresses towards AD dementia. These results could indicate that the drop in discrimination might be linked to the progression of the neurodegenerative process, which occurs differently in males and females.

However, we must note that both the MCI and predominantly AD dementia subgroups comprised much smaller sample sizes than the healthy subgroup (*n* = 13 and *n* = 120 for AD dementia and MCI, respectively, as compared to *n* = 435 for healthy). To investigate whether this could have caused the drop in classifier performance, we conducted a pilot analysis (not shown here), computing an ROC-AUC over an equally small (*n* = 13) sub-sample of healthy subjects. We achieved an AUC that was comparable to the results achieved in our main analysis, indicating that this was not the case. Despite that, our results on the AD dementia subgroup should be interpreted with caution and replicated in more adequately powered AD dementia studies. Additionally, AD dementia patients are older than the other groups, and we reasoned that older age could be driving the loss of discriminatory activity across clinical groups. However, we found no clear effect of increasing age on the ROC-AUC, which suggests that the weakening discriminatory ability of our classifier in the AD continuum is not due to age.

There is evidence that sex-differences occur in clinical manifestation and progression of disease in AD [23]. In particular, women are known to outperform men in neuropsychological tests involving verbal memory; interestingly, this female advantage is largely retained in MCI, in spite of detectable brain damage being equal to that of men [44]. The female advantage is however quickly lost; women affected by MCI have been shown to decline twice as fast as men [45], and therefore later on, at the dementia stage, reach levels similar to those of men, with many studies indicating overall similar cognitive performance in men and women in AD dementia patients (for a review, [23]). In line with this evidence, our results suggest that sex differences fade with disease severity. One might speculate that men and women differ in a number of features at baseline, while these are progressively equalized with the progression of the clinical symptoms over the course of 7 to 10 years. It is likely that specific digital biomarker features in our classifier that discriminate sex differences at baseline (i.e., prior to detectable cognitive symptoms) lose their discriminatory effect at the MCI or AD diagnosis stage. Based on the above, other features collected by Altoida’s application (likely, cognitive related) might become, in later stages of AD, more relevant towards sex classification. Unfortunately, our dataset was too small to test this hypothesis; an analysis of a larger dataset of digital biomarker data collected from Altoida’s application from individuals in MCI and AD dementia stages is needed.

Next, we investigated which features would contribute the most to the performance of our sex classifier in the healthy and MCI population as well as diagnostic subgroups. For the healthy subgroup, we found that the classifier’s discriminatory ability is primarily due to 5 features (Fig. 5B). Interestingly, the most important group of features in the sex classifier seems to be physiological micro tremors recorded by the accelerometer. We observe a higher amplitude of micro tremors at 7–8 Hz as indicative of male subjects. This seems to match findings of [46] where they observed a difference in hand stability with a peak physiological tremor at around 8.3 Hz indicative of males. It also concurs with findings of [47] where steady state hand frequencies led to a sex classification performance of ~ 80% accuracy.

In comparing histogram of acceleration variances between healthy and MCI subjects, we observed that both the males and the females in the MCI have similar feature values as the females in the healthy group. Earlier work showed that differences in force steadiness between sex seem to decline over age [48], which might explain part of this observation as our group of MCI subjects is older than the group of healthy subjects. Yet, we showed in earlier results that the performance of the sex classifier is relatively stable with age. As such, there might be a cognitive basis for the decline in performance of the presented sex classifier. While there is little literature on sex differences in motor ability in AD, considerable evidence has been building in the wider context of neurodegenerative disease, particularly so in Parkinson’s disease. Indeed, a thorough analysis of the individual features might reveal important cognitive components involved, beyond the motor function, for instance, related to attention and visuospatial processing. The clinical relevance of the discriminatory features in our sex classifiers will need to be further examined. An important, unanswered question which follows from our findings, remains if Altoida’s application could also be suited for the discrimination of early signs of other neurodegenerative diseases, such as Parkinson.

In summary, our study provides strong evidence that men and women can be distinguished by an algorithm based on differences in a set of digital biomarkers and that these differences are less prominent in MCI and potentially also in AD dementia. This is consistent with literature on sex-based differential dementia progression. Even if there is literature about sex classifiers based, for example, on EEG data [49] or brain structure [50, 51], the literature on sex classification based on motor pattern is scarcer and is usually focused on walking patterns [52]. Our work stands out as, to the best of our knowledge, no sex-classifier based on visuo-motor digital biomarkers has been tested in a population affected by cognitive impairment, and adds to previous evidence indicating that sex can be detected based on kinematic features alone, in the absence of other demographic features such as height and weight. The fading of classificatory power in a pathologic population is also per se a striking result that has interesting implication about the role of sex in the manifestation of dementia, with potential impact targeted patient monitoring. Our findings are particularly relevant in the context of digital biomarkers, which enable non-invasive and potentially widely applicable predictive diagnostics. Personalization of these tools could further improve their predictive accuracy, allowing for earlier application of targeted preventions, and potentially future disease-modifying treatments.

A limitation of this work is the low number of patients with AD dementia in our sample. Overall, only 2% of our patients had a diagnosis of AD, which limits the statistical power over this subset. In addition, this small subset prevented us from building a sex classifier using only AD subjects and testing that on healthy subjects. Another shortcoming of the data was the imbalance in males and females between the population cohort and the clinical cohort. We overcame this by using sex and cohort stratified data as input to the sex classifiers, yet ideally we would have data equally distributed over age groups. Another shortcoming is that the subject’s sex in the population cohort was self-reported, and a mismatch between gender and sex could lead to noise in the machine learning model. In addition, the fact that cognitive health in the population study was self-reported. Even though these subjects are relatively young to be at risk of Alzheimer’s, they could have other diseases affecting cognition. In general, our data also lacks information on comorbidities such as Parkinson’s.

## Conclusions

In this work, we show that it is possible to differentiate males and females in using digital biomarker data collected from Altoida’s application. The discernible differences seem to decline in subjects with MCI or overt AD, independent of age. In the healthy population, the primary differentiating features are micro hand gestures detectable by Fourier analysis on accelerometer data. We conclude that, akin to what observed with classical biomarkers, sex differences can be observed via digital biomarkers and they have the potential to impact diagnosis and treatment of AD.

Such sex differences, in both classical and digital biomarkers for neurological disorders, are of interest for at least 3 reasons: (1) from a research standpoint, they might shed light on the pathophysiological mechanisms of the disease, which might differ among sexes, with opportunities for personalized treatment; (2) from a predictive medicine perspective, including sex differences might make predictions, especially with algorithms that incorporate multiple variables, more precise (as already done in the Framingham’s risk score [53] for cardiovascular disease, where sex is one of the key variables considered); in particular, considering sex differences might improve our ability to predict fast decliners in MCI patients, which is a key element for planning therapy and care options; (3) from a precision medicine perspective, whether a patient is a man or a woman makes a difference as our data show; more data on sex differences could guide future clinical practice, informing choices for ad hoc prevention (knowing sex-specific risk profiles), diagnosis (adjusting diagnostic cut-offs by sex), and treatment options (if sex specific efficacy and safety profiles will be found). For instance, women have been found to “mask” early stages of Alzheimer [54], hence reaching an MCI diagnosis later than men. Using sex-adjusted tools for diagnosis (or sex-adjusted cut-offs) might be needed to improve diagnostic precision.

Recent research supports the need to account for sex in investigating prospective treatments for AD [43] This should be integrated with the most powerful recent developments in digital medicine to build models of disease development that can fully integrate the effect of sex, digital biomarker technology being one of the most promising tools. The final objective is to build an integrated framework for sex-stratified prediction, monitoring and personalized treatment of neurodegenerative diseases, as supported for example by the Alzheimer’s Precision Medicine Initiative [55]. Such a framework could be used for early detection of the disease, but also to enable targeted prevention strategies and to build personalized treatment strategies. This objective could be achieved by integrating sex with risk stratification based on genetics and individual risk factors, and coupling the process with the extensive use of digital biomarker monitoring applications allowing early detection and treatment of symptoms [56, 57].

It is therefore crucial that the community is aware of such potential differences in order to implement measures to mitigate biases in their clinical applications and ensure precision medicine and precision neurology approaches.

### Conclusions and expert recommendations in the framework of PPPM

The findings presented in this paper add to our body of knowledge and are going to be relevant in the context of predictive, preventive as well as precision medicine in Alzheimer’s patients.

First, sensitive digital biomarkers represent useful tools for personalized prediction of progression from MCI to Alzheimer.

Second, such prediction might in the future allow for individualized follow-up and management of each patient, to reduce risk of progression based on their specific risk profile.

Finally, digital biomarker data might also be used in the future, together with additional biomarkers, in algorithms to identify optimal treatment for each patient.

## Code and data availability

The code and data that support the findings of this study are available from Altoida Inc., but restrictions apply to the availability of these data, which were used under license for the current study, thus they are not publicly available.

## Supplementary Information

Below is the link to the electronic supplementary material.

Supplementary file1 (DOCX 157 KB)
