# Supplementary material for: Digital biomarkers and sex impacts in Alzheimer’s disease management — potential utility for innovative 3P medicine approach
Source: EPMA J. 2022 Jun 6;13(2):299–313. doi: 10.1007/s13167-022-00284-3 (PMC9203627; doi:10.1007/s13167-022-00284-3)
Supplement: Supplementary file 1 — Supplementary file1 (DOCX 157 KB) [file 13167_2022_284_MOESM1_ESM.docx]

Supplementary material

|  |  |  | **Clinical data** | **Population data** | **p-value** |
| --- | --- | --- | --- | --- | --- |
| Number of subjects |  | N (%) | 438 (77%) | 130 (23%) | - |
| Age | Males | Mean (SD) | 69.1 (6.7) | 36.2 (7.6) | <0.001 |
|  | Females | Mean (SD) | 66.7 (6.5) | 32.0 (6.6) | <0.001 |
|  | All | Mean (SD) | 67.6 (6.7) | 35.0 (7.6) | <0.001 |
| Status | Healthy | N (%) | 305 (54%) | 130 (23%) | <0.001 |
|  | MCI ab+ | N (%) | 78 (14%) | 0 (0%) | - |
|  | MCI ab- | N (%) | 42 (7%) | 0 (0%) | - |
|  | AD | N (%) | 13 (2%) | 0 (0%) | - |
| Number of tests (data points) | Males | N | 346 | 602 | <0.001 |
|  | Females | N | 620 | 239 | <0.001 |
|  | All | N | 966 | 841 | <0.001 |
| Number of tests per subject | Males | Median (IQR) | 1 (2) | 7.5 (4) | <0.001 |
|  | Females | Median (IQR) | 1 (2) | 8 (2.25) | <0.001 |
|  | All | Median (IQR) | 1 (2) | 8 (3) | <0.001 |

*Supplementary Table 1. Data characteristics of the clinical and population datasets. P-value is calculated using two-sided t-test for age, chi2 for status and the Mann-Whitney rank test for the number of data points per subject.*

| 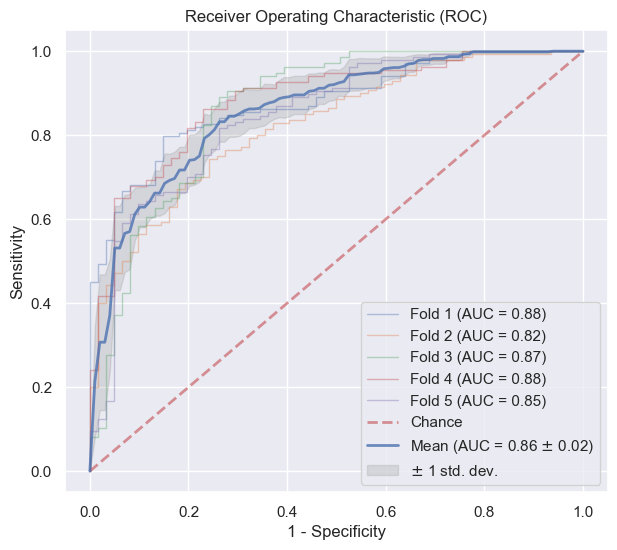  *Supplementary Fig 1. Sex predictor performance using only the subjects from the Japanese population study* | 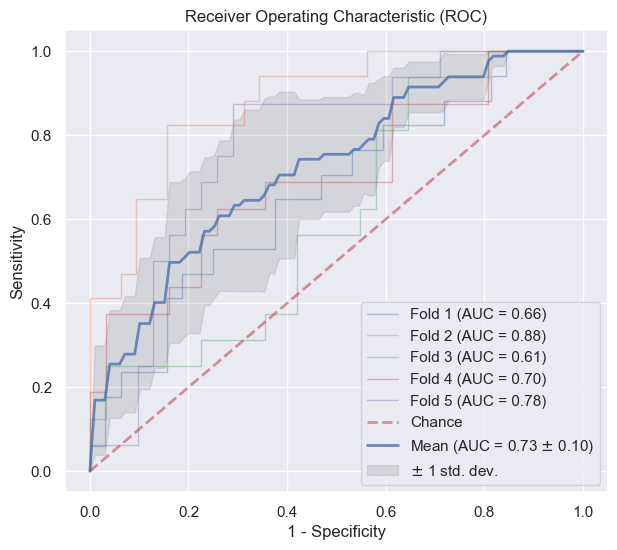*Supplementary Fig 2. Sex predictor performance using only the (elderly) cohort subjects.* |
| --- | --- |
